# Mechanisms Underlying the Induction of Immunological Imprinting by RNA Viruses and Intervention Strategies

**DOI:** 10.3390/v18070745

**Published:** 2026-07-06

**Authors:** Siyu Lin, Guangxu Zhang, Qian Wang, Kun Niu, Qi Liu

**Affiliations:** 1 School of Basic Medicine, Dali University, Dali 671000, China; 20242011040030@stu.dali.edu.cn; 2 Yunnan Key Laboratory of Screening and Research on Anti-Pathogenic Plant Resources from Western Yunnan, Dali 671000, China; 3 Key Laboratory of Medical Molecular Virology (Ministry of Education/National Health Commission/Chinese Academy of Medical Science), Shanghai Institute of Infectious Disease and Biosecurity, School of Basic Medical Sciences, Fudan University, Shanghai 671003, China; gxzhang21@m.fudan.edu.cn; 4 School of Public Health, Dali University, Dali 671000, China

**Keywords:** immune imprinting, memory B cells, influenza virus, coronavirus, dengue virus

## Abstract

The inherent genomic plasticity of RNA viruses, particularly influenza viruses and SARS-CoV-2, poses a major obstacle to the establishment of durable herd immunity. This challenge is further compounded by immune imprinting, whereby prior antigenic exposures bias subsequent responses toward previously encountered epitopes at the expense of effective recognition of antigenically drifted variants. In this review, we delineate the mechanistic basis of immune imprinting, with emphasis on the competitive dominance of cross-reactive memory B cells (MBCs). We discuss how the rapid “back-boosting” of these pre-existing clones can limit de novo priming of naïve B cells—through epitope masking and competition for antigen and T follicular helper cell support—thereby diverting germinal center selection and affinity maturation away from variant-specific de novo epitopes and promoting viral immune escape. To address this challenge, this article further reviews the characteristics of immune imprinting responses in influenza viruses, coronaviruses, and dengue virus, as well as corresponding countermeasures, providing a theoretical basis and new avenues for intervention to address immune imprinting induced by rapidly mutating RNA viruses.

## 1. Introduction

### 1.1. Evolutionary Dynamics and Antigenic Drift of RNA Viruses

The persistence and recurrent epidemics of RNA viruses in host populations are fundamentally attributable to the high variability of their genomes. Unlike DNA viruses, the replication of most RNA viruses relies on RNA-dependent RNA polymerase (RdRp) [1]. Owing to the general lack of 3′–5′ exonuclease proofreading activity, RdRp exhibits low replication fidelity, resulting in the introduction of one nucleotide substitution per 10^4^ to 10^6^ nucleotides in each replication cycle [2]. This high-frequency error-prone replication is not merely a viral defect; rather, it confers upon RNA viruses a survival advantage known as “quasispecies” [3]. A quasispecies is a viral population that does not exist as a single genomic sequence but rather as a mutant spectrum cloud with minor genetic differences distributed around a master sequence [4]. Under the immune pressure of neutralizing antibodies generated during the host–virus antagonistic process, this genetic diversity is rapidly translated into phenotypic adaptation—a process termed antigenic drift [5]. Taking the influenza A virus as an example, the globular heads of its surface hemagglutinin (HA) [6] and neuraminidase (NA) [7] proteins serve as major immunodominant regions that tolerate high-frequency nonsynonymous mutations without compromising viral fitness [8]. A similar mechanism is observed in severe acute respiratory syndrome coronavirus 2 (SARS-CoV-2), where the emergence of the Omicron variant has been accompanied by the accumulation of more than 30 mutations in the receptor-binding domain (RBD) and N-terminal domain (NTD) of its spike (S) protein [9]. These substitutions at key residues alter the conformation of antigenic epitopes, thereby directly diminishing the neutralization capacity of pre-existing host antibodies and enabling the virus to evade the humoral immune barrier established by prior infection or vaccination [10]. This continuous molecular-level evolution constitutes the core driving force behind the repeated breach of host immune defenses by the virus.

### 1.2. Immunological Significance of “Original Antigenic Sin”

The concept of immunological imprinting originated from early observations of the epidemiological characteristics of influenza viruses. In 1960, Thomas Francis Jr. first proposed the hypothesis of “original antigenic sin” (OAS) [11]. By analyzing serological responses to H1N1 influenza virus across different age groups, subsequent studies [12] found that an individual’s immune system appears to be “locked” onto the influenza strain encountered during childhood. When exposed to a new antigenically drifted strain in adulthood, the immune system tends to produce high-titer antibodies against the primary infection strain rather than specific antibodies against the current novel strain [13]. This phenomenon reveals the negative effect of immunological memory under certain contexts, wherein the historical memory of the immune system may interfere with the recognition and response to new antigens. With the continuous advancement of modern immunological techniques, particularly the widespread application of single-cell omics [14], structural immunology [15], and B cell lineage tracing [16], the early concept of “original antigenic sin”—which carried religious metaphorical connotations—has gradually been replaced by the more scientifically descriptive term “immunological imprinting.” Contemporary research has redefined and substantially extended this phenomenon at the molecular and cellular levels.

Immunological imprinting is not merely a bias in antibody specificity; its essence lies in the competitive imbalance between memory B cells (MBCs) and naïve B cells within the germinal center (GC) [17]. When the host is exposed to a novel variant that is similar but not identical to a previously encountered antigen, memory B cells—owing to their lower activation threshold and faster proliferation kinetics [18]—tend to be activated and differentiate into plasma cells preferentially over naïve B cells. However, these “recall-activated” memory cells primarily target conserved epitopes shared between the old and new viruses, rather than the variant-specific epitopes unique to the new virus [19]. This preferential expansion not only generates a wave of high-affinity but poorly neutralizing antibodies against the old strain but may also, through antigen masking [20] and depletion of cytokine resources, inhibit the recruitment and affinity maturation of naïve B cells targeting the novel variant epitopes. Therefore, immunological imprinting can be defined as a conservative strategy in which the immune system tends to leverage pre-existing memory reserves to respond to new threats. This strategy provides rapid protection when viral variation is minor, but under conditions of extensive antigenic drift, it may become a major barrier to the induction of broadly neutralizing antibodies.

## 2. Immunological Mechanisms of Immunological Imprinting Induction

Immunological imprinting is not an inherent design flaw of the immune system, but rather an evolutionary trade-off strategy shaped to cope with recurrent infections [21]. The immune system tends to harness the memory B cell repertoire established by prior antigen exposure: upon re-encounter with a pathogen, the system preferentially mobilizes these existing memory cells to rapidly launch a highly efficient humoral response. Recently, Schiepers et al. [22]. utilized an innovative molecular fate-mapping approach to demonstrate at the single-cell clone level that this “historical preference” is particularly pronounced during homologous boosting. Highly affinity-matured memory B cells rapidly expand and outcompete other cells for microenvironmental resources within the germinal center (GC), resulting in over 90% of the serum antibody response originating from pre-existing clones. Concurrently, the de novo activation and subsequent differentiation of naive B cells targeting newly arising mutant epitopes are severely constrained [23]. In the face of rapidly evolving RNA viruses such as SARS-CoV-2 or influenza A, this evolutionarily conserved survival strategy paradoxically limits the effective induction of broadly neutralizing antibodies at the molecular level. To fully dissect the underlying cues of this phenomenon, this chapter will review the induction mechanisms of immunological imprinting from three dimensions: B cell clonal competition, physical antigen masking, and germinal center microenvironment regulation.

### 2.1. Competitive Advantage of Memory B Cells

**Figure 1:**
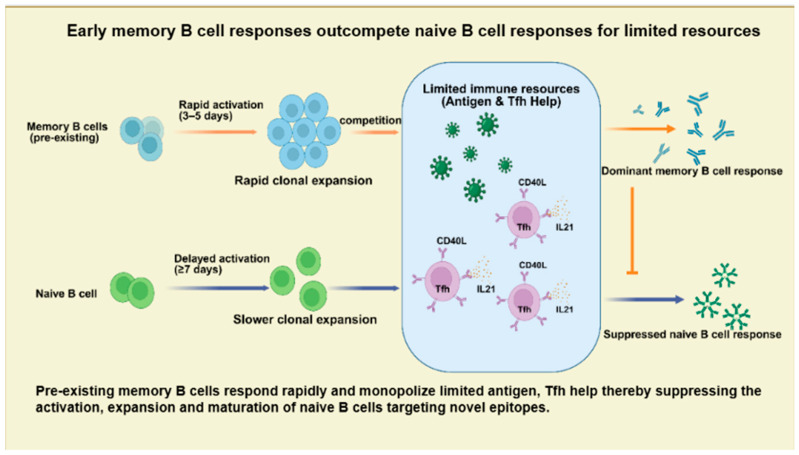
Resource competition between memory B cells and naïve B cells upon re-exposure to a similar viral variant. Pre-existing memory B cells (MBCs) rapidly activate and expand, monopolizing limited antigens and essential signals from follicular helper T (Tfh) cells. This resource depletion suppresses the delayed activation of naïve B cells, hindering effective de novo immune responses against novel viral epitopes. (Created with BioRender.com).

When the host is re-exposed to a similar variant virus, the speed of immune response initiation depends on the kinetic competition between naïve B cells and pre-existing memory B cells [17]. As shown in Figure 1, Memory B cells (MBCs) are intrinsically in a pre-activated epigenetic state [24], with a significantly lower B cell receptor (BCR) signaling threshold compared to naïve B cells. Upon recognition of cognate or cross-reactive antigens, MBCs rapidly differentiate into short-lived plasma cells, producing large quantities of antibodies within 3–5 days post-infection [18]. In contrast, the activation, expansion, and germinal center formation of naïve B cells typically require 7–10 days [25]. This temporal mismatch leads to competitive occupancy of the immune response space by dominant clones. MBCs rapidly deplete limited antigen resources and follicular helper T cell (Tfh) [26] help signals (e.g., IL-21, CD40L), thereby constraining the resources required for the maturation of naïve B cells targeting new epitopes, both physically and in terms of the cytokine environment [27]. Furthermore, reinfection preferentially triggers recall responses against these previously encountered epitopes, such that epitopes that were originally weakly immunogenic or non-neutralizing during primary immunization may become dominant in secondary responses due to the rapid expansion of MBCs [28]. Recent studies in individuals previously exposed to related human coronaviruses and subsequently infected with SARS-CoV-2 have found that, under such circumstances, the memory response may not only be ineffective but can also significantly dampen the activation of B cell responses that would otherwise effectively target novel antigens not present during primary exposure [29], resulting in the production of abundant antibodies with strong binding affinity but poor neutralizing activity.

### 2.2. Epitope Masking and Steric Hindrance

Beyond cellular competition, immunological imprinting manifests at the molecular level as a physical epitope masking effect. Although the surface glycoproteins of variant strains often accumulate numerous mutations for immune evasion, some cross-binding antibodies generated by cross-reactive MBCs can still attach to the viral surface by binding to surrounding conserved regions [30]. Owing to the relatively large molecular weight of antibodies, these non-neutralizing antibodies form a dense protein barrier on the viral surface—a phenomenon known as steric hindrance [31]. This barrier not only prevents newly generated antibodies with potential neutralizing capacity from accessing critical sites but also interferes with BCR recognition and capture of novel antigenic epitopes. Naïve B cells must directly bind antigens via their BCR and undergo endocytic processing in order to present antigenic peptides to T cells and receive signals such as helper cytokines [32]. When key newly mutated sites are masked by pre-existing binding antibodies, naïve B cells targeting these novel sites cannot efficiently take up the antigen, thereby failing to activate clonal expansion of this cell population [33]. This mechanism has been validated in influenza virus models. Angeletti and colleagues found that the stalk domain of influenza virus HA exhibits strong immunogenicity when immunized alone, but B cell responses against the stalk are significantly suppressed in the context of the intact HA molecule. Concurrently, a clinical study by Sun et al. [34]. exploring immunological imprinting in children revealed that the sequence of early influenza exposures severely impairs protective antibody responses against the HA stalk. In a cohort of children primed with H3N2 and subsequently exposed to H1N1, imprinted MBCs preferentially generated abundant cross-binding, non-neutralizing antibodies that recognized the H1N1 stalk but possessed sub-optimal binding affinity. These affinity-deficient, cross-reactive antibodies similarly imposed steric hindrance on the viral stalk, thereby masking critical protective epitopes and depriving peripheral naïve B cells of the opportunity to encounter the novel antigen and initiate activation. Taken together, these findings demonstrate that the physical presence of immunodominant regions (e.g., the HA head) and the pre-existing antibodies they induce can mask relatively concealed, conserved epitopes (e.g., the stalk) through steric hindrance, thereby impeding the effective activation of broadly neutralizing B cells [28]. Collectively, these insights systematically elucidate the molecular underpinnings of immunological imprinting and provide comprehensive evidence for understanding the biological impacts of steric hindrance.

### 2.3. Bias of the Germinal Center Response and the Affinity Trap

The germinal center serves as the training ground for antibody affinity maturation and is also the site where immunological imprinting ultimately becomes “locked in” [35]. High-titer circulating antibodies form immune complexes (ICs) with antigens [36]. Although follicular dendritic cells (FDCs) utilize ICs to select high-affinity B cells [37], under conditions of immunological imprinting [38], these ICs are primarily composed of pre-existing antibodies and antigens from the new virus. This shifts the selective pressure within the germinal center, such that B cells capable of efficiently binding the “pre-existing antibody–new virus” complex are more likely to receive survival signals from FDCs, whereas B cells recognizing novel mutant epitopes are eliminated due to a lack of appropriate antigen presentation [39,40]. Moreover, the capacity of memory B cells to undergo secondary evolution upon entering the germinal center is extremely limited. Studies have shown that most reactivated MBCs tend to differentiate into plasma cells rather than re-enter the GC for somatic hypermutation [41]. Even when a small number of MBCs do enter the GC, their already high affinity for conserved epitopes often allows them to pass selection without further mutation. This results in an affinity trap, wherein the immune system becomes satisfied with pre-existing affinity and prematurely terminates further antibody maturation processes directed against newly mutated sites. This phenomenon is particularly evident in Omicron breakthrough infections, where the expanded clones in patients are predominantly MBCs targeting the original strain, whereas accumulation of de novo somatic hypermutations directed against Omicron-specific mutations is rarely observed [42].

### 2.4. T Cell Memory and the Immunological Imprinting Response

In contrast to the extensive discussion of B cell immunological imprinting, the memory effect at the T cell level exhibits a unique duality. Unlike antibodies that recognize conformational epitopes, T cells recognize linear short peptides that have been degraded and presented by MHC-I molecules [43]. Because the mutation rates of internal proteins of RNA viruses (e.g., nucleocapsid protein N, polymerase components) are much lower than those of surface glycoproteins, CD8+ T cells induced by prior infection generally retain cross-recognition capacity against variant strains. This T cell memory is protective in many contexts; although it does not prevent cellular infection, it rapidly kills infected cells and reduces disease severity [44]. During the COVID-19 pandemic, although Omicron evaded humoral immunity, the relative conservation of cellular immunity explains why severe disease rates did not surge in parallel with infection rates. However, T cells are not without the risk of undergoing an immunological imprinting response. In models such as dengue fever, low-affinity T cells targeting previous strains may produce abundant non-functional cytokines upon reinfection while lacking effective cytotoxic activity, thereby promoting immunopathological damage and even cytokine storms [45]. Upon secondary exposure, memory Tfh cells are more readily activated and rapidly provide helper signals such as CD40L, IL-21, and IL-4 [46]. This help preferentially targets B cells with higher affinity that can more quickly acquire and present similar peptides, thereby creating a memory bias [47] that amplifies the pre-existing antibody response while making it more difficult for naïve B cells corresponding to new epitopes to be activated and expanded.

## 3. Immunological Imprinting Effects in Viral Models

Although immunological imprinting limits the sterilizing immunity of current vaccines against novel variants to some extent, it must be emphasized that existing vaccines continue to play an irreplaceable and critical role in public health. Extensive epidemiological studies comparing vaccinated and unvaccinated populations have consistently demonstrated that vaccination significantly reduces the rates of severe disease, hospitalization, and mortality for both influenza viruses and SARS-CoV-2 [48,49]. The foundational T-cell immunity and cross-reactive antibody networks established by vaccines still provide a robust baseline of protection against severe outcomes. Therefore, discussing immunological imprinting is not to negate the value of current vaccines, but rather to develop next-generation vaccines with broader efficacy upon this solid foundation.

Immunological imprinting is not a product of theoretical deduction but rather an immunological phenomenon established through decades of retrospective cohort studies on infections caused by various RNA viruses. Immunological imprinting can significantly affect vaccine efficacy, as exemplified by the HPV 9-valent vaccine Gardasil 9, highlighting the risk of immune masking in multicomponent vaccines. Gardasil 9 adds five novel antigens to the original quadrivalent vaccine. Studies have observed that individuals previously vaccinated with quadrivalent Gardasil exhibit weaker responses to these five additional antigens when subsequently vaccinated with Gardasil 9, whereas those never vaccinated with the older version mount better responses to the novel antigens upon receiving Gardasil 9. Similarly, in the context of rapidly mutating RNA viruses, the strategy of updating vaccine strains for booster immunization is regarded by the WHO as a standard public health measure. However, clinical data from both seasonal influenza vaccines and COVID-19 boosters consistently indicate that, even with antigenic matching, the immune protection efficacy often falls short of expectations. This effect is largely attributable to the suppression of novel antigen immunogenicity by immunological imprinting. Therefore, this section will further elaborate on the roles and characteristics of the immunological imprinting response at both the individual and population levels in three classical models: influenza virus, coronavirus, and dengue virus.

### 3.1. Immunological Imprinting Effects of Influenza Virus

**Figure 2:**
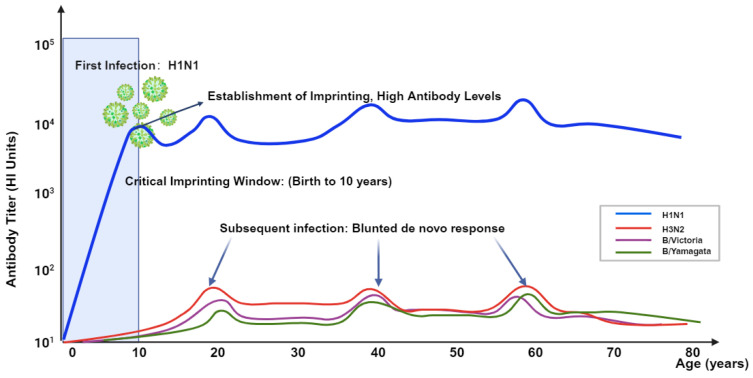
Dynamic model of influenza immune imprinting and the lifelong antibody repertoire. Establishment of immunological imprinting: During the critical imprinting window in early life (typically from birth to 10 years of age, indicated by the light blue shaded area), primary infection with an influenza virus (e.g., hypothetical H1N1 in the figure, thick blue line) elicits a robust germinal center response. This not only establishes extremely high antibody titers against the imprinting strain but also generates long-lived plasma cells (LLPCs) and a durable memory B cell repertoire, maintaining antibody levels at a high baseline throughout the individual’s life. When the individual encounters an antigenically drifted influenza virus or a virus belonging to a different subtype/lineage (e.g., H3N2, B/Victoria, B/Yamagata) later in life, the immune system exhibits an over reliance on prior memory—a phenomenon known as original antigenic sin. Pre existing imprinting memory dominates clonal competition (epitope masking), resulting in severe suppression of the de novo response against the new virus specific epitopes. This is illustrated in the figure by significantly lower peak antibody titers against the new strain upon subsequent infections (Suppressed de novo response). Subsequent heterotypic virus exposure, while eliciting only a weak immune response against itself, typically cross activates immunological memory directed against the imprinting strain. This is visually represented in the graph as synchronous, modest rebound peaks in antibody titers against the imprinting strain (H1N1) at the same ages when new infections occur (e.g., at approximately 20, 40, and 60 years of age). Figures were created with BioRender.com.

Influenza virus, particularly influenza A, provides long-term and detailed epidemiological evidence for immunological imprinting. Its core feature is the “birth cohort effect,” whereby the year of an individual’s birth determines the influenza subtype to which they were first exposed in childhood [50], shaping not only their lifelong immune preferences but also their susceptibility to future pandemic strains [51]. As shown in Figure 2, Epidemiological studies have shown that survivors of the 1918 H1N1 pandemic lacked protection against the 1957 H2N2 pandemic but exhibited unusually low severe disease rates during the 2009 H1N1 outbreak. This phenomenon has been attributed to “HA imprinting” [51]. According to the work of Gostic and colleagues, human immunological imprinting against IAV primarily targets the stalk rather than the head of HA. The HA protein is phylogenetically divided into group 1 (including H1, H2, H5, etc.) and group 2 (including H3, H7, etc.). If an individual’s childhood imprinting is established by a group 1 virus (e.g., H1N1), they will exhibit a certain degree of cross-reactivity as adults against novel avian influenza viruses belonging to the same group (e.g., H5N1); even if this reactivity is non-neutralizing, it can significantly reduce the risk of mortality [51]. In contrast, this imprinting response is almost entirely absent against cross-group viruses (e.g., H7N9 of group 2). Of note, the immunological imprinting effect is not restricted to influenza A but has also been demonstrated in influenza B virus [52]. Although influenza B comprises only two lineages, B/Victoria and B/Yamagata, which diverged only in the 1980s [53], the marked age-specific differences in their distribution are likewise attributable to immunological imprinting. Vieira and colleagues, by analyzing nearly two decades of surveillance data from New Zealand, found that individuals born during the period in the 1990s when B/Yamagata was the exclusively circulating lineage acquired durable and additional protection against B/Yamagata due to primary infection with that lineage, manifested as a significantly lower proportion of B/Yamagata cases in this birth cohort [53]. This finding indicates that the establishment of immunological imprinting does not require a deep evolutionary distance between viruses; even relatively recently diverged lineages, as long as they predominate during a specific period, can leave a lasting immunological imprint on a birth cohort. At the molecular level, antibody responses following influenza vaccination also reveal the negative interference of immunological imprinting. The HA head contains the receptor-binding site and is a major target of potent neutralizing antibodies, yet it evolves extremely rapidly. In contrast, the HA stalk is relatively conserved, and although antibodies induced against it are broadly reactive, they typically exhibit low neutralizing potency [54]. Studies have found that when individuals are repeatedly vaccinated with vaccines that are similar but not perfectly matched to the primary imprinting strain, memory B cells tend to generate “recall antibodies” targeting conserved stalk epitopes. Although these stalk antibodies with weak neutralizing capacity provide a basis for broad protection, high titers of stalk antibodies may physically impede BCR recognition of head-specific epitopes on novel variant strains through steric hindrance [55], resulting in vaccine efficacy that frequently hovers between 40% and 60%. This leads to a clinical paradox: vaccine recipients may appear to have high antibody titers, yet their capacity for immune protection upon challenge with a new strain is extremely limited [56].

### 3.2. Immunological Imprinting Effects of Coronaviruses

Similar to seasonal influenza viruses, coronaviruses that cause common cold symptoms, such as hCoV-NL63, hCoV-229E, and hCoV-OC43, can also induce an immunological imprinting response [57]. Moreover, if an individual has previously been infected with a common cold coronavirus that shares partial antigens with SARS-CoV-2, upon encountering SARS-CoV-2, the immune system may preferentially activate low-affinity memory B cells targeting conserved antigens (e.g., the S2 subunit), thereby suppressing the production of effective neutralizing antibodies against SARS-CoV-2-specific antigens [58]. A longitudinal study of hospitalized COVID-19 patients showed that within 7 days post-infection, cross-reactive antibodies against SARS-CoV-2 S and hCoV-OC43 (a β-coronavirus) S increased significantly, whereas cross-reactive antibodies against hCoV-229E and hCoV-NL63 (α-coronaviruses) did not change markedly [58].

If influenza demonstrates the long-term effects of immunological imprinting, then SARS-CoV-2 has, within just five years, intensively illustrated how immunological imprinting influences individual and population immune responses over a short timescale. With the successive emergence of Omicron and its descendant subvariants, immunological imprinting has become the greatest challenge facing COVID-19 vaccine update strategies [59]. Given that the vast majority of the population has established pre-existing immunity through original strain vaccination or early infection, serological analyses indicate that when these individuals experience Omicron breakthrough infection, their humoral immune responses exhibit strong recall characteristics [60]. Specifically, the antibodies that surge post-infection primarily recognize conserved epitopes shared between WT and Omicron, whereas de novo antibody responses targeting Omicron-specific mutation sites are minimal [60]—a phenomenon termed imprinting suppression of hybrid immunity. Cao and colleagues [38] used deep mutational scanning to demonstrate that after multiple WT vaccinations, the memory B cell repertoire becomes polarized, leading Omicron breakthrough infection to primarily recall cross-reactive clones targeting WT. Although these clones can bind Omicron, their affinity is significantly lower than that for WT, and they are often non-neutralizing [10]. This “ineffective immune focusing” explains why, even after three doses of vaccination, population protection against reinfection with new Omicron subvariants rapidly wanes [38]. Pušnik and colleagues [61] further revealed the depth of this phenomenon through competitive immunization experiments. Their study showed that pre-existing antibodies against SARS-CoV-2 induced by vaccination may develop into original antigenic sin following breakthrough infection with future variant strains, confirming the suppression of novel antigen responses by immunological imprinting. To overcome this issue, the FDA authorized bivalent mRNA vaccines containing the BA.5 component and subsequently the XBB.1.5 monovalent vaccine. However, clinical data indicate that among the neutralizing antibody lineages induced by the bivalent vaccine booster, titers against the original strain remain much higher than those against BA.5 [62]. This suggests that once the immune system is “locked” into an immunological imprinting state by the primary antigen (WT), it is difficult to fully reshape the B cell selection preference of the germinal center, even under stimulation with variant antigens. The immune system tends to expand pre-existing cross-reactive B cell clones that already possess a certain degree of affinity, rather than de novo selecting specific naïve B cells against novel antigens. This “energy-saving strategy” of the immune system becomes a vulnerability in the face of rapidly evolving viruses [63].

If immunological imprinting represents a “conservative strategy” of the host immune system, it conversely becomes a driving force that promotes viral evolution. The pervasive immunological imprinting response in host populations provides a clear selective pressure for the emergence of viral escape mutants, leading to remarkable convergence in viral evolution [64]. When the majority of a population has established pre-existing immunity against a particular strain (e.g., the SARS-CoV-2 original strain or influenza H1N1pdm09) through infection or vaccination, viral mutants capable of evading recognition by these “imprinted antibodies” gain a significant adaptive advantage upon secondary infection. To achieve infection, invasion, and replication, the virus is compelled to mutate at these specific sites to escape this dominant humoral immune pressure [38], creating a selective pressure exerted by pre-existing immunity on variant strains. This selects for mutant lineages that effectively evade cross-reactive antibodies, forming a vicious cycle in which the accumulation of escape mutations further solidifies the imprinting bias of the immune system. This cycle accelerates the pace of viral antigenic drift and renders vaccine strategies based on a single antigen inadequate for long-term control, compelling a rethinking of how to design next-generation immunogens capable of breaking this cycle [65].

### 3.3. Immunological Imprinting of Dengue Virus

**Figure 3:**
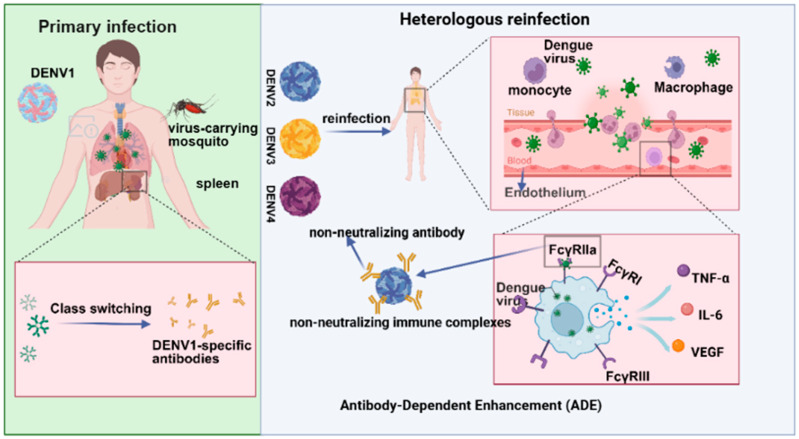
Antibody-dependent enhancement (ADE) during heterotypic dengue virus reinfection. Primary infection with DENV1 induces the production of DENV1 specific antibodies. Upon subsequent reinfection with a heterologous serotype (e.g., DENV2, DENV3, or DENV4), pre existing non neutralizing or subneutralizing antibodies bind to the virus but fail to clear it. Instead, the virus antibody immune complexes are taken up via Fcγ receptors (FcγRI, FcγRIIa, FcγRIII) on monocytes/macrophages, leading to enhanced viral entry and replication. This process triggers the release of pro inflammatory cytokines (e.g., TNF α, IL 6, VEGF), which is associated with increased vascular permeability, recruitment of neutrophils and natural killer cells, and endothelial activation, collectively contributing to the pathogenesis of severe dengue. Figures were created with BioRender.com.

Compared with other RNA viruses, dengue virus not only demonstrates the existence of immunological imprinting but also reveals its potential to induce a severe antibody-dependent enhancement (ADE) effect [66]. As shown in Figure 3, Dengue virus has four serotypes (DENV1-4) [67]. Upon primary infection with one serotype, an individual establishes lifelong immunity against that specific serotype. However, when that individual subsequently experiences a heterotypic infection, immunological imprinting occurs, and the body produces high-titer antibodies against DENV1 rather than specific antibodies against DENV2 [68]. These imprinting-induced cross-reactive antibodies have low affinity for DENV2, are insufficient to neutralize the virus, yet can still bind to viral particles. These “non-neutralizing immune complexes” bind via their Fc region to Fcγ receptors on the surface of monocytes or macrophages, paradoxically facilitating more efficient viral entry into cells and subsequent intracellular replication [68]. The prospective cohort study by Katzelnick and colleagues [68], published in Science in 2017, systematically validated this mechanism in humans for the first time. Through several years of follow-up of children in Nicaragua, the study found that during secondary infection with a heterotypic dengue virus, the risk of severe dengue hemorrhagic fever was significantly elevated, and this risk exhibited a non-linear relationship with pre-existing antibody titers [69]. Within a specific concentration range, these imprinting-induced cross-reactive antibodies not only fail to neutralize the virus but can also, through the formation of immune complexes with the virus and the downstream inflammatory cascade they trigger [70], convert immune protection into immunopathology, leading to a significantly elevated risk of severe dengue hemorrhagic fever or dengue shock syndrome during secondary infection [68]. In dengue hemorrhagic fever models [70], these immune complexes tend to deposit in the alveolar capillary network or glomerular basement membrane, activating the release of C3a and C5a anaphylatoxins and promoting aberrant complement activation. This leads to increased vascular permeability and tissue edema, as well as robust recruitment of neutrophils and macrophages that release pro-inflammatory cytokines such as IL-6 and TNF-α, thereby exacerbating local inflammatory storms and multi-organ dysfunction [71]. Furthermore, the cross-binding antibodies generated by immunological imprinting retain the ability to bind Fc receptors via their Fc region, thereby mediating antibody-dependent cellular cytotoxicity (ADCC) [70]. Moderate ADCC contributes to the clearance of infected cells; however, excessive or aberrant ADCC responses under high viral loads may lead to collateral damage to uninfected bystander cells [72].

## 4. Strategies to Overcome Immunological Imprinting

Immunological imprinting is a phenomenon with profound clinical and public health implications. At the microscopic level, it reshapes the selection of B cell clones; at the macroscopic level, it directly determines the success or failure of vaccination strategies, the trajectory of viral evolution, and the pathological outcomes following infection [73]. To break the host immune system’s dependence on historical antigens, deliberate design is required in terms of antigen engineering, presentation format, and immunization regimens. First, the key to mechanistically overcoming immunological imprinting is to physically block the preferential recognition of non neutralizing, conserved epitopes by memory B cells (MBCs), thereby redirecting immune resources toward critical new mutation sites. Some studies have introduced non natural N glycosylation sites to construct a glycan shield on the surface of conserved epitopes [74]. These glycans not only physically obstruct BCR binding by pre existing MBCs but also, owing to their own weak immunogenicity, avoid inducing new non specific antibodies [75]. For example, in the design of vaccines targeting the F protein of RSV, glycosylation masking of non neutralizing epitopes successfully increased neutralizing antibody titers by several fold [74]. Beyond antigen design, the timing and modality of immunization schedules are also critical factors in breaking the imprinting response. Heterologous vaccination using multiple platforms [76] is one immunological strategy to overcome imprinting; different vaccine platforms, such as adenovirus vectors (Ad5/ChAdOx1) and mRNA, exhibit distinct antigen presentation kinetics and adjuvant effects [77]. Heterologous boosting—for instance, priming with an inactivated or adenovirus vaccine followed by an mRNA booster—can introduce new Tfh cell epitope helper signals, thereby activating a repertoire of naïve B cell clones distinct from those engaged during primary immunization [78]. Mesin and colleagues similarly found that even during homologous boosting, secondary germinal centers are populated predominantly by naïve B cells lacking prior germinal center experience, rather than by memory B cells [79]. This indicates that the capacity of memory B cells to enter germinal centers is intrinsically limited, providing a theoretical basis for resetting the direction of immune responses through immunization schedule interventions. Furthermore, extending the booster interval (e.g., to 6–12 months) allows the germinal center (GC) reaction to mature fully, permits plasma cell homing to the bone marrow, and enables circulating antibody levels to decline naturally. Vaccination at this time not only reduces the risk of rapid antigen clearance by “old antibodies” but also awakens deep memory B cells that have undergone extensive somatic hypermutation and possess broader recognition capabilities. Tas and colleagues [80] also found that increasing the immunization dose—through repeated or high dose antigen injections—can deplete peripheral circulating antibodies, thereby partially overcoming antibody mediated constraints on B cell recruitment. However, the effect of repeated injections in overcoming antibody blockade is not without limitations, being directly related to the affinity and concentration of pre existing antibodies. Wan and colleagues proposed an “epitope hierarchy reshaping” strategy. Using a sequential immunization approach in ferrets, they found that an antigenically distant PAN/99 strain significantly enhanced viral clearance efficiency and reduced total viral shedding [81]. In the context of immunological imprinting, naïve B cells face strong competition from MBCs. To reverse this disadvantage, potent adjuvants that significantly lower the activation threshold of naïve B cells can be used. Traditional aluminum adjuvants primarily induce Th2 type responses, whereas next generation adjuvants, particularly oil in water emulsion adjuvants, have shown excellent performance in responding to influenza pandemics and enhancing vaccine responses in the elderly and children. One such adjuvant, MF59 [82], is composed of squalene, Tween 80, and Span 85. It acts by inducing the release of local chemokines such as CXCL10 at the injection site, recruiting large numbers of antigen presenting cells (e.g., macrophages and dendritic cells) to the site, thereby markedly enhancing the immune response. Another adjuvant, AS03, incorporates α tocopherol in addition to squalene and Tween 80 [83]. Clinical data indicate that both AS03 and MF59 induce broader cross neutralizing antibodies in influenza vaccines [84]. Clinical studies have demonstrated that unadjuvanted vaccines tend to recall only pre existing stalk antibodies—those generated by immunological imprinting of conserved regions. In contrast, the incorporation of AS03 adjuvant not only enhances overall immunogenicity but also promotes the recruitment of B cells targeting novel epitopes, successfully shifting the response after a second booster from the induction of highly conserved HA2 stalk antibodies toward the HA1 head of entirely new strains, thereby generating protective antibodies with high neutralizing capacity and high affinity. The strong innate immune signals provided by adjuvants upregulate co stimulatory molecules (CD80/86) and cytokine receptors on B cells, enabling naïve B cells to become activated and enter germinal centers even when they bind antigen with low affinity [85].

To address the severe antibody-dependent enhancement (ADE) effect induced by specific virus models (such as the dengue virus), intervention strategies must be more precise. Future vaccine development must not only ensure a perfect balance in the immunogenicity of the four serotype antigens in tetravalent vaccines to avoid immune bias driven by dominant serotypes; it can also involve mutating viral envelope proteins at the molecular level (e.g., eliminating the highly conserved fusion loop epitope). This reduces the induction of non-neutralizing cross-reactive antibodies, thereby fundamentally blocking the enhanced infection mediated by the binding of these antibodies to Fcγ receptors on the surface of monocytes and macrophages. From the broader perspective of global medical countermeasures for pandemic preparedness, establishing rapidly iterable mRNA vaccine platforms combined with advanced multivalent antigen co-display technologies, such as mosaic nanoparticles, will serve as a critical line of defense in overcoming pre-existing immunological imprinting in populations and achieving broad-spectrum protection.

Notably, artificial intelligence (AI) and other advanced computational biology technologies are providing revolutionary tools to overcome immunological imprinting. By analyzing massive genomic datasets through machine learning algorithms, researchers can predict the evolutionary trajectories and potential escape mutations of RNA viruses in advance. Integrating AI-based protein structure prediction and design tools (such as AlphaFold), scientists can now precisely design engineered novel antigens in silico. These antigens can intelligently mask conserved epitopes prone to triggering imprinting responses, while simultaneously stabilizing and exposing key neutralizing epitopes that are otherwise difficult to induce naturally. This cutting-edge strategy, fusing AI computation with structural immunology, holds the promise of fundamentally reshaping our reactive paradigm in vaccine design against rapidly mutating viruses.

In clinical practice, the novel dengue vaccine TAK-003 (QDENGA) provides a compelling example of addressing the risks of immunological imprinting and ADE. Unlike earlier vaccines that faced risks of enhanced severe disease, TAK-003 is constructed on an attenuated DENV-2 backbone and expresses antigens from all four serotypes. Clinical data have confirmed that it demonstrates robust overall protective efficacy in both baseline seronegative and seropositive populations, substantially reducing the risk of hospitalization [86]. By optimizing the vaccine backbone to balance multivalent immune responses, TAK-003 largely mitigates the pathogenic ADE induced by immunological imprinting, providing important practical support for actively evading imprinting responses through vaccine design.

## 5. Conclusions and Future Perspectives

Immunological imprinting embodies the tradeoff made by adaptive immunity between speed and precision. On one hand, memory B cells induced by prior infection or vaccination can rapidly expand upon re exposure and generate early antibody peaks, thereby shortening the viral replication window and reducing the risk of severe disease. On the other hand, in the face of RNA viruses with pronounced antigenic drift, this recall response tends to focus on conserved, weakly neutralizing epitopes shared among variant strains. Together with epitope masking and germinal center selection bias, this response impairs the de novo recruitment and affinity maturation of B cells targeting critical newly mutated sites. The ultimate clinical manifestation is breakthrough infection by further mutated variants and suboptimal vaccine efficacy following vaccine strain updates. At the population level, pre-existing dominant antibodies against SARS-CoV-2 can also exert immune selection pressure on the virus, driving convergent evolution of escape mutations and accelerating antigenic drift. Immunological imprinting describes the biased process of B cell clonal selection, whereas antibody dependent enhancement (ADE) describes the functional consequence of the antibodies generated by this bias. Not all immunological imprinting leads to ADE; however, in the dengue virus model, immunological imprinting is a prerequisite for ADE to occur. This unique example serves as a warning that the design of immunization strategies against multi serotype viruses must be approached with extreme caution, so as to avoid inducing cross reactive antibodies that fall into the low affinity ADE trap [44].

To overcome the dilemma posed by immunological imprinting, the development of targeted novel intervention strategies is particularly urgent. As explored in this review, future countermeasures should focus on breaking the bias of B cell clonal selection. At the antigen design level, glycan shields can be introduced to mask conserved epitopes, thereby redirecting immune resources toward novel critical mutation sites. Regarding immunization regimens, adopting heterologous sequential vaccination or appropriately extending the interval between booster doses can help overcome the competitive suppression by memory B cells, promoting the recruitment and maturation of naïve B cells against novel epitopes. Furthermore, incorporating novel potent adjuvants, such as oil-in-water emulsions, can effectively lower the activation threshold of naïve B cells. These novel intervention concepts not only provide a solid theoretical foundation for addressing the immune evasion induced by rapidly mutating RNA viruses but also point the direction for the development of next-generation, broad-spectrum universal vaccines.

## Data Availability

Not applicable.
